# Impact of fatty pancreas and lifestyle on the development of subclinical chronic pancreatitis in healthy people undergoing a medical checkup

**DOI:** 10.1186/s12199-019-0763-2

**Published:** 2019-02-07

**Authors:** Makoto Fujii, Yuko Ohno, Makoto Yamada, Yoshihiro Kamada, Eiji Miyoshi

**Affiliations:** 1 Department of Molecular Biochemistry and Clinical Investigation, Osaka University Graduate School of Medicine, 1-7 Yamadaoka, Suita, Osaka 565-0871 Japan; 2 Department of Mathematical Health Science, Osaka University Graduate School of Medicine, 1-7 Yamadaoka, Suita, 565-0871 Osaka Japan; 3 aMs New Otani Clinic, 1-4-1 Shiromi Chuo-ku, Osaka, Osaka Japan

**Keywords:** Pancreas, Fat accumulation, Ectopic fat, Chronic pancreatitis, Cohort study

## Abstract

**Background:**

Although fat accumulation in human organs is associated with a variety of diseases, there is little evidence about the effect of a fatty pancreas on the development of subclinical chronic pancreatitis over the clinical course.

**Methods:**

We conducted a prospective cohort study from 2008 to 2014 of patients who underwent a medical checkup consultation for fat accumulated in the pancreas. Patients included in the analysis were divided into a non-fatty pancreas group (*n* = 9710) and fatty pancreas group (*n* = 223). The primary end point was the odds ratio (OR) for chronic pancreatitis associated with fatty pancreas, which was diagnosed using ultrasonography. We used a multiple logistic regression model to estimate the OR and the corresponding 95% confidence interval (CI).

**Results:**

Ninety-two people were diagnosed with chronic pancreatitis, including both presumptive and definitive diagnoses. Twelve people were diagnosed with chronic pancreatitis by ultrasonography among the 223 patients with fatty pancreas, and 80 patients among 9710 were diagnosed with non-fatty pancreas. The crude OR was 6.85 (95% CI 3.68, 12.75), and the multiple adjusted OR was 3.96 (95% CI 2.04, 7.66).

**Conclusions:**

Fat accumulation in the pancreas could be a risk factor for developing subclinical chronic pancreatitis.

## Introduction

Pancreatic cancer has a 5-year relative survival rate of 9.2% in Japan, and patients with this cancer have a poor prognosis [1]. It is not known whether chronic pancreatitis is a risk factor for pancreatic cancer [2–4]. Although heavy drinking is an important etiology for chronic pancreatitis, only a small portion of drinkers develop chronic pancreatitis. The population rate of chronic pancreatitis is different between studies because the diagnosis is limited by the invasiveness of pancreatic biopsy [5]. Recently, we have found fatty degeneration and fibrosis in pancreatic tissue surrounding most cases of pancreatic adenocarcinoma [6]. Interestingly, similar pathological changes in the pancreas were observed by autopsy of patients with pancreatic diseases, suggesting there may be many more patients with subclinical chronic pancreatitis. Furthermore, we have demonstrated fatty acid-mediated stromal reprograming of pancreatic stellate cells, leading to pancreatic inflammation and fibrosis [7]. Several studies on the association between non-alcoholic fatty pancreas disease (NAFPD) and metabolic syndrome have been performed, and ectopic fat was shown to cause various diseases such as pancreatic cancer and insulin resistance [8–10]. Thus, ectopic fat and its relationship with diabetes and metabolic syndrome have been studied, but the association between changes in ectopic fat and development of chronic pancreatitis in clinical course has not been investigated [11]. Most previous research was performed using retrospective studies in patient populations with a genetic predisposition. Therefore, it is necessary to identify factors that are associated with fatty pancreas and chronic pancreatitis development for an early detection and prevention of pancreatic cancer using a prospective study. In the present study, we have investigated whether or not fatty changes in the pancreas are a risk factor for the development of chronic pancreatitis in a prospective study.

## Materials and methods

### Study design

This study was designed as a medical examination-based cohort study.

### Participant selection

**Fig. 1:**
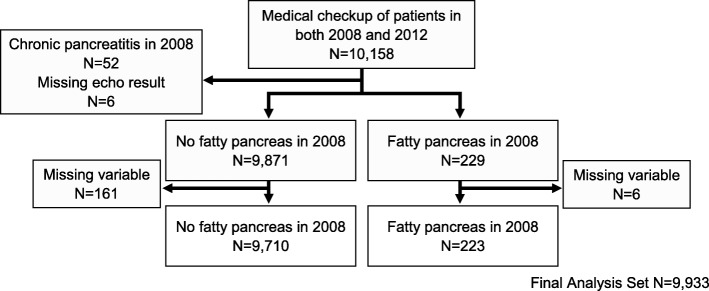
Flow chart of participant selection

The participants in the study were relatively healthy individuals who elected to undergo a medical checkup with abdominal ultrasonography at aMs New Otani Clinic (Osaka, Japan) during the study period. There were 25,897 people in 2008 and 30,188 people in 2014 who underwent this examination. Of the 25,897 people examined in 2008, 10,158 were re-examined in 2014. The recruitment of large numbers of participants is necessary to be able to quantify the incidence of rare diseases, such as chronic pancreatitis. The study was designed to determine whether participants in 2008 had findings consistent with chronic pancreatitis in 2014. In 2008, 52 people were diagnosed previously with chronic pancreatitis and they were excluded from the analysis. There were 173 patients with missing data who were excluded from the analysis. The final analysis included 9933 people as shown in Fig. 1.

### Ethics approval

In this study, all data were de-identified. This study was approved by the ethics committee at the Osaka University Hospital (approval number 13563(260-2)-5).

### Outcome measures

The main outcomes of interest in this study were the subsequent chronic pancreatitis rates. An odds ratio (OR) was used to evaluate adiposity and its relationship with chronic pancreatitis and fat accumulation of the pancreas.

### Diagnostic criteria for chronic pancreatitis

The Japanese Pancreatic Society (JPS) provided two levels of diagnosis for chronic pancreatitis, “definite chronic pancreatitis” and “probable chronic pancreatitis.” “Pancreatic stones,” one of the diagnostic criteria of chronic pancreatitis shown by JPS, satisfies the criteria for diagnosing definite chronic pancreatitis. The JPS described that a diagnosis of probable chronic pancreatitis was made when “intrapancreatic coarse hyperreflectives, irregular dilation of pancreatic ducts or pancreatic deformity with irregular contour” were met [12–14]. The JPS discussed that patients with probable chronic pancreatitis were started on therapy before the definite diagnosis was established by follow-up studies. Because our study was based on a medical examination, and screening was considered important, we classified 92 subjects who met two levels of chronic pancreatitis. In this study, only a small number of cases were obtained, with 42 and 50 patients displaying definite chronic pancreatitis and probable chronic pancreatitis, respectively.

### Definition of fatty pancreas and diagnostic criteria for fatty pancreas

The definition of fatty pancreas differs among previous studies, and there is no generally accepted definition [9, 15]. The normal echogenicity of the pancreas is similar to that of the normal liver [16]. However, as fat accumulates in the pancreas, it becomes more highly echogenic [17]. Therefore, we defined fatty pancreas ultrasonographically by the presence of higher echogenicity in the pancreas than in the left lobe of the liver, when both were viewed using the same US window [18].

### Medical checkup data

Baseline data including sociodemographic variables such as age and sex; basic variables such as height, weight, body mass index (BMI), systolic blood pressure (SBP), and diastolic blood pressure (DBP); medical history variables such as diabetes (DM), stroke, kidney disease, and anemia; laboratory test results such as aspartate aminotransferase (AST), alanine aminotransferase (ALT), gamma-glutamyltransferase (GGT), amylase, total cholesterol (TC), triglycerides (TG), high-density lipoprotein (HDL), low-density lipoprotein (LDL), fasting blood sugar level (FBS), and hemoglobin A1c (HbA1c); medications such as antihypertensives (AHTs), oral hypoglycemic agents (OHAs), and lipid-lowering drugs (LLDs); and lifestyle factors such as the amount of alcohol consumed, Brinkman index (the number of cigarettes smoked per day multiplied by the number of years of smoking), weight gain, exercise, physical activity, walking speed, weight cycling, eating before bedtime, eating after dinner, no breakfast, and sufficient sleep were obtained using a self-administered questionnaire that was partially supported and reconfirmed by a personal interview with physicians or nurses. All data obtained were entered into a database. We included all outliers when the data obtained in our study were analyzed.

Smoking status was considered a non-smoker if the Brinkman index was 0; a light smoker had a Brinkman index < 0 and < 200; a middle smoker had a Brinkman index of ≥ 200 and < 400; and a heavy smoker had a Brinkman index of ≥ 400. Some variables based on previous studies were used for the analysis in combination with other variables. Ethanol intake was categorized into four groups as 0 g/week, 1–139 g/week, 140–279 g/week, or ≥ 280 g/week on the basis of the standard established by the Ministry of Health, Labour and Welfare of Japan [19]. The LDL level was estimated using the Friedewald formula as follows:

$$ \mathrm{LDL}=\mathrm{TC}-\mathrm{HDL}-\mathrm{TG}/5.0. $$

During the study period, the standardization program of HbA1c measurement was changed from the Japan Diabetes Society (JDS) standard to a national glycohemoglobin standardization program (NGSP) using an international standard. The HbA1c (%) obtained in 2008 was estimated as an HbA1c (NGSP) equivalent value (%) and calculated using the following formula:

$$ \mathrm{HbA}1\mathrm{c}\ \left(\mathrm{NGSP}\right)\ \left(\%\right)=\mathrm{HbA}1\mathrm{c}\ \left(\mathrm{JDS}\right)\ \left(\%\right)+0.4\%. $$

The pulse pressure (PP) was estimated using the formula:

$$ \mathrm{PP}=\mathrm{SBP}-\mathrm{DBP}. $$

### Statistical analysis

The participants were classified according to the combination of the pancreas echo inspection status and drinking level. We compared baseline characteristics among the two groups using an analysis of variance (ANOVA), the Aspin–Welch test, the chi-square test, or Fisher’s exact test. We calculated the effect sizes including Cohen’s *d* for continuous variable and Cramer *V* (phi coefficient) for categorical variables. The OR was estimated using a logistic model. The 95% CI of the OR was calculated using the Wald confidence interval.

First, a linear model was determined. Second, a univariate analysis was conducted to find the OR, and then, a multivariable-adjusted OR was estimated. Multivariable logistic regression models were prepared to estimate the risk of chronic pancreatitis associated with potential predictors including sociodemographic variables; clinical variables such as alcohol exposure, smoking exposure, and laboratory data; and lifestyle factors. Inclusion of variables in the models was based on existing knowledge of risk factors for chronic pancreatitis [20–22]. The condition index was provided from weighting the linear regression using the diagonal element of the weight matrix used in computing the Hessian matrix, which was used to check for multicollinearity [23–26]. Additionally, variables with tolerance less than 0.1 or variables with variance inflation greater than 10 were judged to have multiple collinearity. Predictive and complexity characteristics of the model were considered during modeling. Evaluation criteria of variable selection were used as well as the Akaike information criterion and Bayesian information criterion. Evaluation of the goodness of fit and significance of model measures for logistic regression were used as well as fractional polynomials, the Hosmer–Lemeshow test, the area under the curve of the receiver operating characteristic curve, the Pearson test, and the deviance goodness-of-fit tests [27, 28].

Finally, we examined the confounding factors. Interaction terms of the likely explanatory variables associated with fatty pancreas was included in the model. Statistical significance was assessed using the 95% CI, and *P* < 0.05 was considered significant. All *P* values were two-tailed. All data were statistically analyzed using SAS statistical software (version 9.4; SAS Institute Inc., Cary, NC).

## Results

In the final analysis, 9933 subjects were included and classified into the fatty pancreas (*n* = 223, 2.24%) and normal pancreas (*n* = 9710, 97.76%) groups. The cumulative incidence rate was 185.24 per 100,000 (male, 284.81; female, 82.02). Table 1 compares the baseline characteristics of the participants who underwent the examination in both years and those who were not re-examined in 2014, with regard to pancreatic status. In participants with normal pancreases, there were significant differences between those who were and those who were not re-examined with regard to age, sex, BMI, SBP, PP, AST/ALT, GGT, LDL/HDL, FBS, HbA1c, and Brinkman index. The effect sizes for all the variables were ≤ 0.18. There were significant differences in sex and BMI between participants and non-participants with fatty pancreas, but no differences in other parameters. The effect sizes for all the variables were ≤ 0.31. In addition, the data in Table 1 permit a comparison of the baseline characteristics of participants with normal pancreases and those with fatty pancreases in the participants in 2014. There were significant differences in age, sex, BMI, SBP, PP, AST/ ALT, GGT, LDL/HDL, FBS, HbA1c, and Brinkman index between participants who had fatty pancreas and those who did not. However, there was no significant difference in alcohol intake between these groups. The effect sizes with regard to BMI and age were 0.69 and 0.64, respectively, and the others were < 0.6.

**Table 1:** Comparison of the baseline characteristics of participants and non-participants in 2014, classified according to the presence or absence of fatty pancreas

	Normal pancreas				Fatty pancreas		Difference Participants only^‡^			
	Participants	Non-participants			Participants	Non-participants				
Variable	(*n* = 9710)	(*n* = 13,058)	*P* value*	ES^†^	(*n* = 223)	(*n* = 2283)	*P* value	ES	*P* value	ES
Sex (male/female), *n*	4898/4812	5894/7164	< 0.0001	0.05	158/65	709/1574	< 0.0001	0.23	< 0.0001	0.06
Age, mean (SD)	46.62 (8.66)	47.45 (9.80)	< 0.0001	0.09	52.1 (9.26)	51.9 (8.56)	0.7992	0.02	< 0.0001	0.64
Body mass index, mean (SD)	22.23 (3.03)	22.79 (3.32)	< 0.0001	0.18	24.29 (3.18)	25.38 (3.59)	< 0.0001	0.31	< 0.0001	0.69
Systolic blood pressure, mean (SD)	109.09 (14.41)	110.01 (14.48)	< 0.0001	0.06	115.27 (14.50)	116.06 (14.51)	0.4378	0.05	< 0.0001	0.43
Pulse pressure, mean (SD)	44 .06 (8.76)	44.47 (8.92)	0.0003	0.05	45.97(8.72)	46.34 (9.45)	0.5733	0.04	0.0010	0.22
AST/ALT, mean	1.15 (0.36)	1.12 (0.37)	< 0.0001	0.08	1.02 (0.31)	0.97 (0.32)	0.0576	0.16	< 0.0001	0.36
Gamma-glutamyltransferase, mean (SD)	35.93 (46.82)	38.28 (48.23)	0.0002	0.05	52.2 (61.11)	51.0 (57.08)	0.7699	0.02	0.0001	0.35
LDL/HDL, mean (SD)	1.96 (0.70)	2.01 (0.73)	< 0.0001	0.07	2.21 (0.69)	2.32 (0.75)	0.0366	0.15	< 0.0001	0.37
Fasting blood glucose, mean (SD)	97.06 (13.97)	98.51 (15.92)	< 0.0001	0.10	104.3 (21.93)	105.01 (21.55)	0.6405	0.03	< 0.0001	0.56
Hemoglobin A1c, mean (SD)	5.55 (0.50)	5.59 (0.56)	< 0.0001	0.07	5.83 (0.83)	5.81 (0.76)	0.8582	0.03	< 0.0001	0.58
Alcohol intake										
None, *n* (%)	6596 (67.93)	8756 (67.05)	0.2138	0.01	134 (60.09)	1442 (63.16)	0.6718	0.03	0.0770	0.03
1–139 g/week, *n* (%)	1003 (10.33)	1348 (10.32)			25 (11.21)	250 (10.95)				
140–279 g/week, *n* (%)	1377 (14.18)	1871 (14.33)			37 (16.59)	370 (16.21)				
≤ 280 g/week, *n* (%)	734 (7.56)	1083 (8.29)			27 (12.11)	221 (9.68)				
Smoking										
Brinkman index = 0, *n* (%)	7719 (79.50)	9898 (75.80)	< 0.0001	0.06	168 (75.34)	1711 (74.95)	0.3189	0.04	0.0060	0.04
0 < Brinkman index < 200, *n* (%)	321 (3.31)	1925 (14.74)			4 (1.79)	92 (4.03)				
200 ≤ Brinkman index < 400, *n* (%)	505 (5.20)	588 (4.50)			8 (3.59)	95 (4.16)				
400 ≤ Brinkman index, *n* (%)	1165 (12.00)	647 (4.95)			43 (27.12)	385 (16.86)				

*AST/ALT* serum aspartate aminotransferase to alanine aminotransferase activity ratio, *LDL/HDL* serum low-density lipoprotein to high-density lipoprotein ratio

*Aspin–Welch test, chi-square test, or Fisher’s exact test

^†^ES: Effect size obtained using Cohen’s *d* with continuous variables or Cramer *V* and phi with categorical variables

^‡^Difference between participants with normal pancreas and those with fatty pancreas among participants in 2014

Table 2 summarizes the multivariable-adjusted OR of fatty pancreas in patients with chronic pancreatitis. We used a logistic model without adjustment for GGT, because GGT was strongly correlated with ethanol intake. Twelve people were diagnosed with chronic pancreatitis among the 223 fatty pancreas patients, and 80 people were diagnosed with chronic pancreatitis among the 9710 normal pancreas subjects. The crude OR was 6.85 (3.68, 12.75), and the multiple adjusted OR was 3.96 (2.04, 7.66).

**Table 2:** Multivariable-adjusted odds ratio for the development of chronic pancreatitis in participants with fatty pancreas

	Chronic pancreatitis	No chronic pancreatitis	Univariate analysis *n* = 9933		Multivariable analysis* n = 9933				
	*n* = 92	*n* = 9841	Crude OR with 95% CI	AUC	Adjusted OR with 95% CI	Tolerance	VIF	Condition index	AUC
Fat accumulation									
Normal pancreas, *n* (%)	80 (0.82)	9630 (99.18)	Reference	0.555	Reference	0.982	1.018	3.139	0.802
Fatty pancreas, *n* (%)	12 (5.38)	211 (94.62)	6.846 (3.676, 12.750)		3.957 (2.043, 7.664)				
Demographics									
Age, mean (SD)	54.72 (9.68)	46.67 (8.67)	1.095 (1.073, 1.118)	0.728	1.099 (1.072, 1.127)	0.821	1.218		
Women, *n* (%)	20 (0.41)	4857 (99.59)	Reference	0.638	Reference	0.600	1.667		
Men, *n* (%)	72 (1.42)	4984 (98.58)	3.508 (2.134, 5.767)		1.943 (1.065, 3.542)				
Body mass index, mean (SD)	22.93 (2.77)	22.27 (3.05)	1.067 (1.004, 1.133)	0.575	0.971 (0.890, 1.059)	0.668	1.500		
Systolic blood pressure, mean (SD)	115.7 (15.04)	109.17 (14.42)	1.027 (1.015, 1.039)	0.629	0.999 (0.978, 1.020)	0.429	2.329		
Pulse pressure, mean (SD)	47.39 (9.83)	44.07 (8.75)	1.038 (1.018, 1.060)	0.605	1.010 (0.980, 1.042)	0.529	1.889		
Laboratory data									
AST/ALT, mean (SD)	1.05 (0.33)	1.15 (0.36)	0.436 (0.232, 0.820)	0.581	0.444 (0.203, 1.001)	0.728	1.374		
Gamma-glutamyltransferase, mean (SD)	56.01 (68.97)	36.11 (46.96)	1.004 (1.002, 1.006)	0.644					
LDL/HDL, mean (SD)	2.03 (0.69)	1.96 (0.7)	1.131 (0.850, 1.504)	0.529	0.878 (0.633, 1.220)	0.772	1.295		
Fasting blood glucose, mean (SD)	101.55 (13.47)	97.18 (14.24)	1.011 (1.004, 1.019)	0.631	0.999 (0.979, 1.019)	0.391	2.558		
Hemoglobin A1c, mean (SD)	5.72 (0.49)	5.55 (0.51)	1.424 (1.140, 1.779)	0.611	0.972 (0.557, 1.697)	0.403	2.480		
Lifestyle factors									
Ethanol intake, *n* (%)									
None, *n* (%)	46 (0.68)	6684 (99.32)	Reference	0.605	Reference	0.765	1.307		
1–139 g/week, *n* (%)	10 (0.97)	1018 (99.03)	1.427 (0.718, 2.837)		0.971 (0.476, 1.977)				
140–279 g/week, *n* (%)	18 (1.27)	1396 (98.73)	1.874 (1.083, 3.241)		1.084 (0.597, 1.971)				
≤ 280 g/week, *n* (%)	18 (2.37)	743 (97.67)	3.520 (2.031, 6.102)		1.933 (1.023, 3.652)				
Smoking, *n* (%)									
Brinkman index = 0, *n* (%)	55 (0.7)	7832 (99.3)	Reference	0.607	Reference	0.856	1.169		
0 < Brinkman index < 200, *n* (%)	2 (0.62)	323 (99.38)	0.882 (0.214, 3.631)		1.415 (0.337, 5.936)				
200 ≤ Brinkman index < 400, *n* (%)	9 (1.75)	504 (98.25)	2.543 (1.250, 5.175)		3.529 (1.657, 7.517)				
400 ≤ Brinkman index, *n* (%)	26 (2.15)	1182 (97.85)	3.132 (1.957, 5.014)		1.922 (1.145, 3.224)				

*OR* odds ratio, *95% CI* 95% interval estimates with confidence limits, *AUC* area under the curve, *VIF* variance inflation, *AST/ALT* aspartate aminotransferase to alanine aminotransferase ratio, *LDL/HDL* low-density lipoprotein to high-density lipoprotein ratio

*Adjusted for age, sex, body mass index, systolic blood pressure, pulse pressure, AST/ALT, LDL/HDL, log-transformed fasting blood glucose concentration, log-transformed hemoglobin A1c, alcohol intake status, and Brinkman index

Diagnoses of chronic pancreatitis were made in the following groups of smokers: 26 people among the 1208 heavy smokers, nine people among the 513 middle smokers, two people among the 325 light smokers, and 55 people among the 7887 non-smokers. The multiple adjusted OR for heavy smokers was 1.92 (1.15, 3.22); in middle smokers, the OR was 3.53 (1.66, 7.52); and in light smokers, the OR was 1.42 (0.34, 5.94).

For chronic pancreatitis, 18 of the 761 people who had an ethanol intake ≥ 280 g/week were diagnosed with chronic pancreatitis, as were 18 of the 1414 people who had an intake of 140–279 g/week, 10 of the 1028 people with an intake of 1–139 g/week, and 46 of the 6730 people who did not drink alcohol. The multiple adjusted OR for the ≥ 280 g/week group was 1.93 (1.02, 3.65); for the 140–279 g/week group, it was 1.08 (0.60, 1.97); and for the 1–139 g/week group, it was 0.97 (0.48, 1.98).

The condition index was 3.14, which was provided in collinearity diagnostics analysis. All tolerances were greater than 0.39, and all variance inflations were less than 2.56. We found no evidence of multicollinearity.

We followed standard methods to examine over-fitting for multiple logistic regression. The area under the curve in the final model was 0.802. The interaction was not observed because the *P* value of the interaction terms between the fatty pancreas and other covariates in a multivariate logistic regression analysis was 0.29 or more (Table 3). We found no evidence of interaction between alcohol intake and smoking (*P* = 0.847). All *P* values by the Hosmer–Lemeshow goodness-of-fit test in multivariate logistic regression model including interaction terms were over 0.64. A marked reduction in the fitness of the multivariate logistic regression model, including interaction terms, was not observed (Table 3).

**Table 3:** Examination of the interactions in the multivariate logistic regression model

	*P* value for interaction term	HL* goodness-of-fit test		
		Chi-square	DF	*P* value
Fatty pancreas*drinking	0.610	5.973	8	0.650
Fatty pancreas*Brinkman index	0.846	5.129	8	0.744
Fatty pancreas*age	0.287	4.634	8	0.796
Fatty pancreas*body mass index	0.502	4.619	8	0.797
Fatty pancreas*sex	0.358	6.037	8	0.643

*DF* degrees of freedom

*Hosmer–Lemeshow goodness-of-fit test

## Discussion

In this prospective cohort study, the main findings showed that, among the participants, fat accumulation in the pancreas was a significant risk factor for chronic pancreatitis. The findings might be important for early detection of high-risk groups of pancreatic cancer. To our knowledge, this is the first study to report the effect of fat accumulation on the incidence of chronic pancreatitis as a perspective study. Additionally, smoking, with a Brinkman index over 200, was found to be associated with an increased risk of chronic pancreatitis.

Comparisons of the baseline characteristics of the 9933 subjects of this study and the 15,341 who did not undergo re-examination in 2014 showed significant differences in all the parameters except alcohol intake in people with normal pancreases. In the smaller group of people with fatty pancreas, significant differences were only detected in age and BMI. However, the effect sizes were very small in all cases, implying that the effect of selection bias in this study was small.

The term “pancreatic lipomatosis” was first coined by Ogilive [29], but it has been replaced by “steatosis.” Previous studies have shown that fat accumulation is associated with male sex, age older than 60 years, BMI, fatty liver, hyperlipidemia, metabolic syndrome, higher insulin resistance, visceral fat area, triglyceride and ALT levels, and alcohol consumption [9, 16, 17, 30–36]. Past studies have shown that chronic pancreatitis is associated with smoking [37–41]. However, previous studies have not shown that fatty pancreas is associated with chronic pancreatitis [32]. Moreover, an association between chronic pancreatitis and fatty pancreas has never been demonstrated in large cohort studies. Most of these studies had a small sample size, and the subjects were highly selected for investigation, such as “scheduled for endoscopic ultrasound” or “visited the obesity clinic,” which limits the generalizability of their results. Thus, the research design was almost always a cross-sectional study or a case–control study. Our study, however, recruited healthy people prospectively and followed them up. With a significantly large cohort study, we observed that the cumulative incidence of chronic pancreatitis was 926 per 100,000, which may be considered to be a reliable estimate for the general population. Additionally, we found that fatty pancreas, BMI, male sex, age, and smoking were independently associated with chronic pancreatitis.

There are only a few studies on pancreatic steatosis, and its pathophysiological mechanisms remain largely unknown [42]. Many previous studies have indicated that chronic high-fat diets increased pancreatic free fatty acids and lipid peroxidation, which is associated with pancreatic injuries and collagen synthesis by activated pancreatic stellate cells, and induce oxide injuries and fibrogenesis of pancreatic cells in rats [43–46]. Similar to findings in rats, fat accumulation in the human pancreas may be a risk factor for fibrosis, including acute and chronic inflammation, which is consistent with the results of this study.

There was no significant relationship between BMI and chronic pancreatitis, which is consistent with the results of a previous study [40, 47]. The average BMI of participants in this study was 24.29 in the fatty pancreas group and 22.3 in the normal pancreas group, both much lower than participants in previous studies in other countries [16, 17, 30–32, 40, 47]. The East Asian race, including the Japanese, is a race with a low BMI worldwide [48]. In this study, there was no significant difference between BMI at 2008 and 2014 in any of the groups, and it was considered that there is little bias resulting from excessive obesity.

For smoking, the odds of chronic pancreatitis were higher for patients with a Brinkman index > 200, and this result was also consistent with numerous previous studies [38, 40]. However, the smoking rate in this study was about 20%, which is lower than in previous studies and lower than the Japanese national average of 28.2%. However, it was meaningful that the risk of smoking for chronic pancreatitis was also obtained, which is consistent with the results of a previous study [38, 40].

Regarding the drinking of alcohol, the odds ratio increased with an increase in alcohol consumption. The odds ratio of chronic pancreatitis was higher for patients consuming over 280 g/week, while no significant difference was seen for other categories; this finding was consistent with previous studies [40, 47, 49]. The proportion of drinkers in this study was around 35%, which is lower than that reported for other countries as well as for the Japanese national average of 44.6% [50]. Thus, the results of the present study may be dependent on subjects with relatively healthy habits.

A strength of this large-scale and prospective study was that it was possible to obtain patients with the onset of chronic pancreatitis and low morbidity. The 5-year cumulative incidence of chronic pancreatitis calculated here included “probable” cases and was therefore considerably higher than that reported in previous studies, but the 5-year cumulative incidence of definitively diagnosed chronic pancreatitis was 30.21 per 100,000, which is slightly lower than that previously reported [41, 51].

In Japan, the incidence of chronic pancreatitis was reported to be increasing, and a larger cohort study is needed [49]. Additionally, it was possible to examine factors such as fatty pancreas, smoking, and drinking. In cohort studies with a small sample size, it is difficult to accurately determine the incidence of pancreatitis, so we assumed that it was not reported accurately. Moreover, a pancreatic biopsy is difficult to obtain, and it relies on diagnostic imaging compared with diseases in other organs. Abdominal ultrasonography is superior to the other methods because it is noninvasive and comparatively simple to use. Fat accumulation can be identified at a relatively early stage, and further research could lead to large-scale screening.

There are some limitations to this study. First, data were obtained during single examinations conducted in 2008 and 2014, and therefore, the time to onset of chronic pancreatitis could not be included in the analysis. Even if an echo image of chronic pancreatitis is obtained, it is not always obtained again in the next fiscal year. Therefore, it is difficult to accurately obtain information on when pancreatitis occurred. Second, sociological and economic variables, such as income, educational level, or literacy, were not included in our models to consider their possible effects on chronic pancreatitis because we did not have these data in our database. However, to the best of our knowledge, no previous studies have suggested any role for these variables in the incidence of chronic pancreatitis. Third, few patients exhibited definite chronic pancreatitis in this study; therefore, the overall relative risk of pancreatitis may be higher than that estimated in the current work. Participants in this study represented a relatively healthy group. In such participants, it is possible that the risk of chronic pancreatitis may be lower than the general population. However, it is difficult to implement many cohort studies targeting groups of a larger scale than this research, and our current results provide valuable insight. In the future, we will continue to follow-up the participants, and a detailed prospective study will be required to address the above limitations. Although similar results for some risk factors have been reported, external validity concerning fat accumulation in organs requires additional studies.

## Conclusion

The present study shows that fat accumulation in the pancreas is a significant risk factor for chronic pancreatitis. Evaluation of pancreatic fat accumulation using abdominal ultrasonography provides a noninvasive screening strategy for high-risk groups of chronic pancreatitis, a condition which may lead to pancreatic cancer in future.

## Abbreviations

AHTs: Antihypertensives
ALT: Alanine aminotransferase
ANOVA: Analysis of variance
AST: Aspartate aminotransferase
BMI: Body mass index
CI: Confidence interval
DBP: Diastolic blood pressure
DM: Diabetes
FBS: Fasting blood sugar level
GGT: Gamma-glutamyltransferase
HbA1c: Hemoglobin A1c
HDL: High-density lipoprotein
JDS: Japan Diabetes Society
JPS: The Japanese Pancreatic Society
LDL: Low-density lipoprotein
LLDs: Lipid-lowering drugs
NAFPD: Non-alcoholic fatty pancreas disease
NGSP: National glycohemoglobin standardization program
OHAs: Oral hypoglycemic agents
OR: Odds ratio
PP: Pulse pressure
SBP: Systolic blood pressure
TC: Total cholesterol
TG: Triglycerides
